# Der Rolle der DNA-Schadensantwort bei granulomatösen Erkrankungen

**DOI:** 10.1007/s00393-022-01260-y

**Published:** 2022-08-25

**Authors:** Lea A. R. Fabry, Antigoni Triantafyllopoulou

**Affiliations:** 1 Medizinische Klinik m.S. Rheumatologie und Klinische Immunologie, Charité – Universitätsmedizin Berlin, Charitéplatz 1, 10117 Berlin, Deutschland; 2 Deutsches Rheuma Forschungszentrum, ein Institut der Leibniz Gemeinschaft, Berlin, Deutschland

**Keywords:** Granulome, Multinukleäre Makrophagen, DNA-Schadensantwort, Genotoxischer Stress, Sarkoidose, Granulomas, Multinucleated macrophages, DNA damage response, Genotoxic stress, Sarcoidosis

## Abstract

Granulome sind organisierte Aggregate von Immunzellen, die sich infolge eines persistierenden Stimulus bilden und bei verschiedenen rheumatischen Erkrankungen zu finden sind. Zentraler Bestandteil von Granulomen ist eine Vielzahl unterschiedlicher Makrophagensubtypen. Darunter befinden sich auch multinukleäre Makrophagen, die mehrere Zellkerne aufweisen. Die genauen Mechanismen, welche die Granulomentstehung vermitteln, sind bislang noch nicht vollständig aufgeklärt. Neuere Daten zeigen jedoch, dass die DNA-Schadensantwort eine relevante Rolle bei der Entstehung multinukleärer Makrophagen und damit bei der Bildung von Granulomen spielen könnte.

## Abstract (en)

Granulomas are organized aggregates of immune cells, which are formed in response to a persistent stimulus and are found in various rheumatic diseases, including sarcoidosis, rheumatoid arthritis and granulomatosis with polyangiitis. The core of granulomas contains a multitude of different macrophage subtypes, including multinucleated macrophages and foam cells. The mechanisms which induce the formation of granulomas are not well understood; however, recent data show that the DNA damage response regulates granuloma macrophage differentiation.

## Granulomatöse Erkrankungen in der Rheumatologie

Kennzeichen granulomatöser Erkrankungen ist das Auftreten von Granulomen. Diese sind organisierte Aggregate von Immunzellen [1], die als Folge eines persistierenden Stimulus entstehen [1–4]. Der Stimulus kann infektiöser Genese sein [5] wie z. B. bei Tuberkulose [3, 4]. Aber auch in einer Reihe rheumatischer Erkrankungen wie der rheumatoiden Arthritis [6], Granulomatose mit Polyangiitis [7], Sarkoidose [1, 8] und Riesenzellarteriitis [9] kann man das Auftreten von Granulomen beobachten. Bei einigen dieser Erkrankungen ist die Granulombildung bedeutsamer Bestandteil der Pathogenese, indem gesundes Gewebe durch Granulomgewebe verdrängt und dadurch eine Funktionsstörung verursacht [10, 11] wird. Auch bei der Diagnostik sind Granulome von klinischer Relevanz. So kann der histopathologische Nachweis eines Granuloms [6] zur Diagnose einiger granulomatöser Erkrankungen beitragen [7, 9].

## Granulome zeigen eine Vielfalt verschiedener Makrophagen

Granulome stellen eine Ansammlung einer Vielzahl unterschiedlichster Makrophagentypen dar [3, 4, 12, 13]. Darunter sind Epitheloidmakrophagen [12], durch Lipidtröpfchen gekennzeichnete Schaumzellen [3, 4] und Makrophagen, die eine unterschiedliche Anzahl an Zellkernen aufweisen (multinukleäre Makrophagen) [3, 13]. Diese Granulommakrophagen unterscheiden sich in ihrer Funktion [14], ihren genetischen Programmen [3] sowie in ihrem Phänotyp und Metabolismus [15–17] deutlich von den Makrophagen, welche in gesundem Gewebe vorkommen [3]. Die genauen Mechanismen, welche die Differenzierung der unterschiedlichen Granulommakrophagentypen vermitteln, sind bislang nur unvollständig untersucht und verstanden, scheinen jedoch entscheidend für den Krankheitsverlauf zu sein [3, 4, 18, 19].

## Multinukleäre Makrophagen

Polyploide Makrophagen sind ein Subtyp von Granulommakrophagen, welche sich in Granulomen entwickeln und eine variable Anzahl von Kopien ihres Genoms [3] in sich tragen. Aber auch unter homöostatischen Bedingungen sind Makrophagen dazu in der Lage, mehrkernige Zellen zu bilden. Ein Beispiel dafür sind Osteoklasten, ein spezialisierter multinukleärer Makrophagentyp, der am Knochenstoffwechsel beteiligt ist und durch Zell-Zell-Fusion von Makrophagenvorläufern entsteht [20]. Anhand dieses Paradigmas für die Entstehung multinukleärer Makrophagen wurde vermutet, dass der Mechanismus der Zell-Zell-Fusion auch für die Entstehung von Granulommakrophagen verantwortlich sein könnte [13]. Eine neuere Studie [3] hat jedoch gezeigt, dass auch andere Mechanismen zur Entstehung polyploider Makrophagen führen können. Dabei wird der DNA-Schadensantwort („DNA damage response“ [DDR]) eine wichtige Rolle zugeschrieben [3].

## Die DNA-Schadensantwort dient zum Erhalt der genomischen Integrität

**Figure 1:**
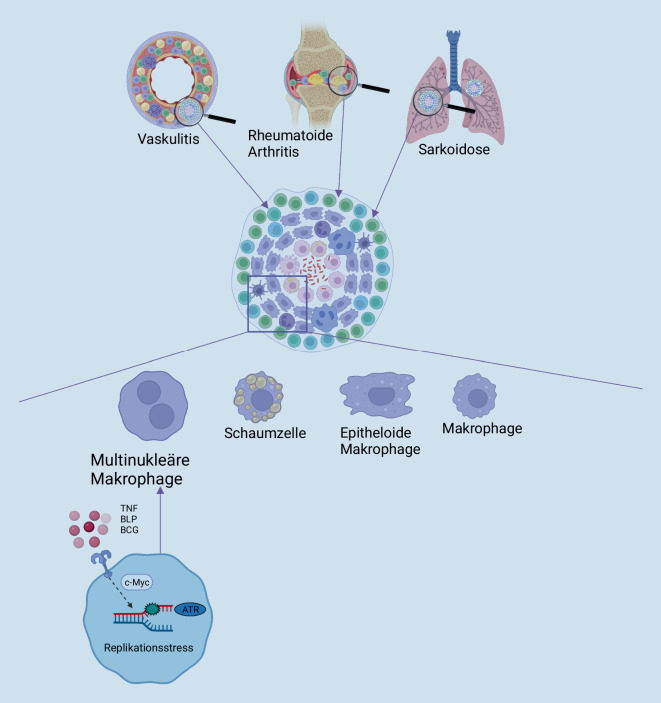

Die DNA-Schadensantwort (Abb. 1 und 2) ist ein Netzwerk komplexer Mechanismen, welches dafür sorgt, dass die genomische Integrität gewahrt und auf Schädigungen der DNA adäquat geantwortet wird [21]. Die DDR ähnelt dem Aufbau einer klassischen Signalkaskade, welche aus Sensoren, Transduktoren und Effektoren besteht [22]. Bei der DDR dient jedoch nicht, wie bei vielen Signalkaskaden, ein Ligand, welcher einen Rezeptor bindet, als Anstoß für den Ablauf der Kaskade [23]. Vielmehr kommt es aufgrund eines DNA-Schadens zu einer Veränderung der Struktur der DNA [24]. Diese strukturelle Veränderung wird von den Sensorproteinen erkannt, welche dafür sorgen, dass die Transduktoren zur Stelle der DNA-Schädigung rekrutiert werden und eine Aktivierung der DDR-Kaskade stattfindet [23, 25, 26]. Die Transduktoren können ihrerseits direkt [27–29] oder indirekt über Mediatoren [30, 31] dafür sorgen, dass ihre Substrate, die Effektoren, ebenfalls den Ort der Schädigung erreichen und durch eine Reihe von Modifikationen [32–35] aktiviert werden, um dem Schaden adäquat zu begegnen [4, 23, 31].

**Figure 2:**
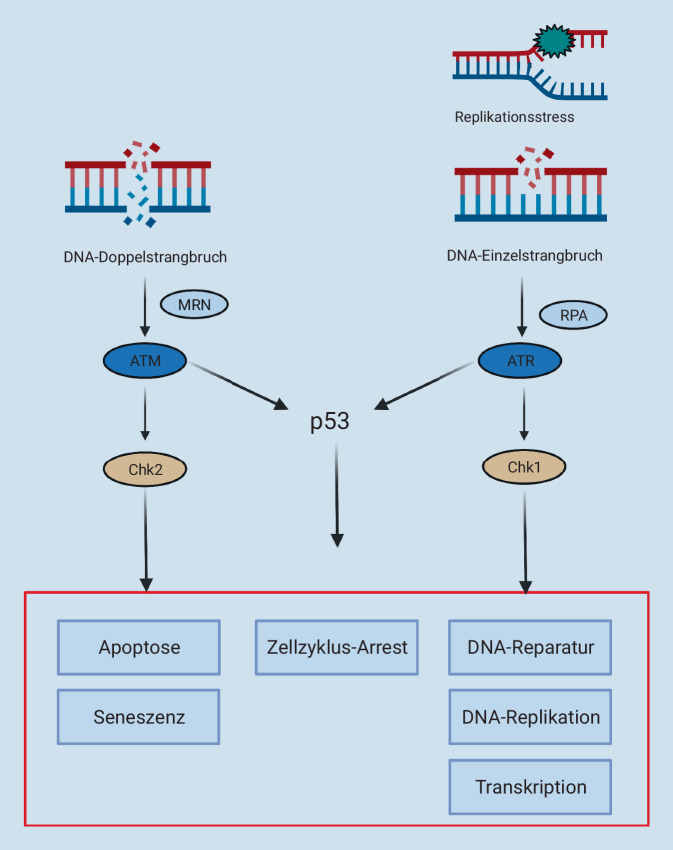

Die bekanntesten Transduktoren der DNA-Schadensantwort sind die Phosphatidylinositol 3‑Kinase-ähnlichen-Proteinkinasen (PIKK) ATM („ataxia teleangiectasia mutated kinase“) und ATR („ataxia telangiectasia and Rad3-related kinase“) (Abb. 2; [36, 37]). Beide Enzyme haben teilweise überschneidende, teilweise verschiedene Aufgaben bei der Reparatur von DNA-Schädigungen und können miteinander interagieren, um sich gegenseitig zu unterstützen [23, 38]. Während ATM v. a. durch DNA Doppelstrangbrüche aktiviert wird, reagiert ATR auf ein ganzes Spektrum an DNA-Schädigungen [23]. Neben der Vermittlung der Reparatur von Einzel- und Doppelstrangbrüchen spielt ATR eine wichtige Rolle bei der Antwort auf Replikationsstress (RS) [3, 4, 39], einem Zustand, bei dem das Fortschreiten der Replikationsgabel behindert und die DNA-Synthese verlangsamt wird [3, 4, 40].

Entsteht ein Doppelstrangbruch der DNA, wird dieser durch den Sensorproteinkomplex MRN, bestehend aus MRE11, Rad50 und NBS1, erkannt [26]. MRN kann mit ATM interagieren und dessen Aktivierung vermitteln [41], sodass die Signalkaskade gestartet wird. ATM sorgt seinerseits dafür, dass die Checkpointkinase 2 (Chk2) phosphoryliert wird [42]. Diese Aktivierung kann dazu beitragen, dass der „Wächter des Genoms“, p53, stabilisiert wird [43]. p53 initiiert eine Reihe von Reaktionen, die zum Arrest des Zellzyklus und der Reparatur des DNA-Schadens führen können [44]. Liegt ein irreparabler Schaden vor, dann kann p53 ein Seneszenzprogramm oder einen programmierten Zelltod (Apoptose) auslösen [45, 46]. Auch ATR ist in der Lage, p53 zu aktivieren. Entstehen Strangbrüche oder tritt Replikationsstress auf, dann wird die vorliegende einzelsträngige DNA von Replikationsprotein A (RPA) gebunden [47]. RPA vermittelt die Rekrutierung von ATR mit seinem assoziierten Protein, „ATR-interacting protein“ (ATRIP), [48] an die Stelle der Läsion [47]. ATR wird aktiviert und kann Checkpointkinase 1 (Chk1) phosphorylieren [31] und ebenfalls die Aktivierung von p53 vermitteln [49, 50].

Die DNA-Schadensantwort ist somit essenziell, um Fehler, die während der Replikation entstehen und durch endogene oder exogene Noxen ausgelöst werden können [4, 31], auszubessern oder die Zelle ggf. an weiterer Replikation und Teilung zu hindern [45, 46]. Zusammen mit einer Reihe weiterer Regulationsmechanismen, die dafür sorgen, dass der Zellzyklus reguliert und Mitose und Replikation stets alternieren, trägt die DNA-Schadensantwort dazu bei, dass der diploide Zustand der meisten Säugetierzellen erhalten bleibt [3, 4, 51]. Doch nicht nur unter physiologischen Bedingungen spielt die DDR eine relevante Rolle. Untersuchungen zeigen, dass Mutationen in der DNA-Schadensantwort die Entstehung von Krebs begünstigen können [52].

## Die DNA-Schadensantwort hat einen Dosiseffekt bei der Entstehung von Krebs

Genomische Instabilität ist ein Merkmal, welches viele Krebszellen teilen [53]. Durch Onkogenmutation wird vermehrte Proliferation gefördert, und DNA-Schädigungen und Replikationsstress treten gehäuft auf [54]. Zusätzlich wurde beobachtet, dass die „replication stress response“ (RSR) in vielen Krebszellen hochreguliert ist – ein Phänomen, welches man in gesunden Zellen mit einer hohen Proliferationsrate bisher nicht beobachtet hat [55]. Diese Betrachtung führte zu der Vermutung, dass die DNA-Schadensantwort eine essenzielle Rolle für das Überleben der Krebszellen hat [47].

Eine Untersuchung, bei welcher der Verlust eines ATR-Allels [56] erzeugt wurde, hat gezeigt, dass ATR als haploinsuffizienter Tumorsuppressor fungiert [56]. Dies bedeutet, dass Zellen, in welchen ein funktionales ATR-Allel fehlt, nicht dazu in der Lage sind, die physiologischen zellulären Funktionen auszuüben, welche die Tumorentstehung verhindern [57]. Dadurch zeigt sich bei Mäusen mit einer heterozygoten Mutation von ATR, ein erhöhtes Tumorrisiko [56]. Ein vollständiges genetisches Ausschalten hingegen führt dazu, dass das Tumorrisiko verringert wird [58].

Daraus lässt sich eine duale Rolle des DDR-Signalwegs für die Entstehung und das Voranschreiten von Tumoren ableiten: Im physiologischen Ausgangszustand können DDR und RSR das Risiko der Krebsentstehung verringern, indem die genomische Integrität gewahrt wird [21]. Im Fall der Aktivierung eines Onkogens, dient die DDR als letzte Barriere, um den Wandel einer präkanzerösen Läsion in eine kanzeröse Läsion zu verhindern [4, 31]. P53 wird dabei eine wichtige Rolle zugeschrieben [3, 4, 31]. Wird diese Schwelle jedoch ebenfalls überschritten, können Onkogene Replikationsstress auslösen, was zu einer Aktivierung der RSR und DDR führt [54, 59]. Die darauffolgende Aktivierung von ATR und Chk1 reduziert den RS [39] und ermöglicht es dem Tumor, mit dem erhöhten genotoxischen Stress umzugehen [55]. In diesem Szenario zeichnet sich ein Dosiseffekt der DDR ab. Die DDR ist mit den entstehenden Schäden überfordert und schafft es nicht, diese vollkommen zu reparieren und die Tumorzellen zu eliminieren. Sie ist jedoch gerade ausgeprägt genug, um den Krebszellen die Toleranz der entstehenden Schädigungen zu ermöglichen und die Apoptose dieser zu verhindern. Eine ähnliche Rolle könnte die DDR bei der Entstehung multinukleärer Makrophagen in granulomatösen Erkrankungen spielen.

## Entstehung polyploider Makrophagen durch chronische Aktivierung der DNA-Schadensantwort

Makrophagen entstehen aus Vorläuferzellen, die entweder dem Dottersack, der fötalen Leber oder dem Knochenmark entstammen. Über die Blutgefäße können sie verschiedene Gewebe erreichen. Die im Gewebe vorhandenen Einflüsse, können daraufhin spezifische Programme in den Makrophagenvorläuferzellen auslösen und die Differenzierung zu Gewebsmakrophagen einleiten [60]. Bei entzündlichen Prozessen kann das Gewebe mit Zytokinen, Wachstumsfaktoren und anderen entzündungsfördernden Faktoren angereichert sein, welche einen Stimulus für die Entstehung eines Granuloms darstellen können [4]. Diese inflammatorische Umgebung kann die Differenzierung von Makrophagenvorläufern zu Makrophagen beeinflussen und zur Bildung von multinukleären Granulommakrophagen führen [3]. Multinukleäre Makrophagen unterscheiden sich neben ihrer Polyploidie auch in der Genexpression [3] von den unter homöostatischen Bedingungen entstehenden Makrophagen. Dies deutet darauf hin, dass durch die in der entzündlichen Umgebung vorliegenden Stimuli ein nichtkanonisches Differenzierungsprogramm [3] in den Makrophagen ausgelöst wird.

**Figure 3:**
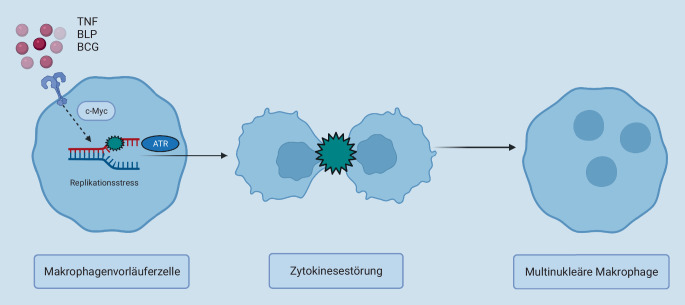

Stimulationen von Makrophagen mit dem Zytokin Tumornekrosefaktor (TNF), mit bakteriellem Lipoprotein (BLP) und Infektion mit Bacillus Calmette-Guérin (BCG) zeigten, dass durch diese Faktoren ein Programm ausgelöst wird, welches, anders als in Osteoklasten, nicht durch Zellfusion, sondern durch Zytokinesestörungen oder Überspringen der Mitose zur Entstehung polyploider Makrophagen führt (Abb. 3; [3]). Dabei wurde beobachtet, dass in den Makrophagen vermehrt Replikationsstress auftritt. Dieser führt zu mitotischen Defekten und verursacht dadurch die Entstehung von mononukleären Makrophagen mit einem tetraploiden Zellkern, binukleären Makrophagen oder Makrophagen mit multiplen Nuclei. Der Replikationsstress und die dadurch eingeleitete RSR scheinen von c‑Myc, einem Transkriptionsfaktor, dessen Aktivität bzw. Expression in vielen Tumoren dysreguliert [61] und mit Replikationsstress assoziiert ist [62], abhängig zu sein [3]. Anders als in vielen Tumoren, bei welchen p53 Mutationen aufweist und dadurch funktionell verändert ist [63], ist der „Wächter des Genoms“ bei der Entstehung polyploider Makrophagen aktiviert [3]. Dennoch hemmt p53 trotz seiner Aktivität die Formation und Proliferation von polyploiden Makrophagen nicht [3]. Dies ist möglicherweise durch eine Umgehung der kanonischen p53-Kaskade durch Wachstumsfaktorsignalwege zu erklären [3, 64].

Eine mögliche Erklärung für die Entstehung multinukleärer Makrophagen in chronischer Entzündung könnte also darin bestehen, dass die Stimulation mit entzündungsfördernden Faktoren in den Makrophagen ein genetisches Programm aktiviert, bei welchem Gene, die mit RS assoziiert sind, verstärkt exprimiert werden. In der Folge treten vermehrt RS und DNA-Schädigungen auf, die Defekte in der Mitose verursachen und zur Entstehung polyploider Makrophagen führen. Diese Formation multinukleärer Makrophagen ist abhängig von der DNA-Schadensantwort.

## Rolle der DNA-Schadensantwort in Makrophagen bei rheumatischen Erkrankungen

Granulome treten bei verschiedenen rheumatischen Erkrankungen auf. Dazu gehören Sarkoidose [8], rheumatoide Arthritis [6] und Vaskulitiden [7, 9]. Ob die Granulombildung vorteilhaft oder schädlich für den Betroffenen ist, lässt sich nicht eindeutig sagen. Bei rheumatoider Arthritis und Sarkoidose scheint es sich um pathologische Mechanismen zu handeln, bei denen Granulomgewebe gesundes Gewebe verdrängt und dadurch eine Funktionsbeeinträchtigung zur Folge haben kann. Tuberkulosegranulome galten lange Zeit als protektive Strukturen, die dazu beitragen, eine Verbreitung von Mykobakterien zu verhindern [65]. Mittlerweile ist es jedoch umstritten, Granulome bei Tuberkulose ausschließlich als schützend zu betrachten [65]. Umso wichtiger ist es, die Entstehung und Funktion von Granulomen genauer zu untersuchen. Herrtwich et al. führten zunächst verschiedene In-vitro-Versuche durch, um die Mechanismen, welche der Granulombildung unterliegen, besser zu verstehen [3]. Dabei fiel auf, dass die DDR insbesondere bei Stimulation mit TNF aktiviert ist [3]. Dies könnte darauf hindeuten, dass die DDR bei der Bildung von Granulomen in TNF-abhängigen rheumatischen Erkrankungen wie Morbus Crohn [66], rheumatoider Arthritis [67] und Sarkoidose [68] eine Rolle spielt. Um herauszufinden, ob sich diese Beobachtungen tatsächlich in rheumatischen Erkrankungen widerspiegeln, wurden Biopsien von Patienten mit Tuberkulose, Riesenzellarteriitis und Sarkoidose untersucht [3]. Dabei fiel auf, dass in den Granulomarealen, welche vermehrt multinukleäre Makrophagen enthalten, eine gesteigerte Expression von Proteinen, die mit RS assoziiert sind, zu finden ist [3]. Diese Observationen gemeinsam mit den Daten einer Genexpressionsanalyse von Tuberkulosegranulomen, welche eine Hochregulation von DDR-Genen zeigt [3], unterstützen die Vermutung, dass die DDR wichtig für das Auftreten multinukleärer Granulommakrophagen in rheumatischen Erkrankungen ist. Ob das Auftreten von DDR und RS als Marker für einen progressiven Krankheitsverlauf von granulomatösen Erkrankungen unterschiedlicher Genese genutzt werden könnte, ist eine spannende Frage, die intensiv erforscht wird.

## Fazit/Ausblick für die Zukunft

Die Entstehung und Funktion von Granulomen in rheumatischen Erkrankungen werfen noch immer eine Reihe von Fragen auf. Trotz des Wissens, dass die Granulombildung durch einen persistierenden Stimulus eingeleitet werden kann [1–4], ist der genaue mechanistische Vorgang [69], welcher zur Formation der Immunzellen in einem organisierten Aggregat [1] führt, bislang nicht aufgeklärt worden. Zudem stellt die Frage nach der Funktion von Granulomen noch viele Forscher vor ein Rätsel. Haben Granulome eine protektive Wirkung und sollte man deren Entstehung daher fördern? Oder könnte eine Hemmung der Entwicklung von Granulomen den Krankheitsverlauf für den Patienten verbessern? Das Wissen um die Relevanz der DNA-Schadensantwort für die Entstehung multinukleärer Granulommakrophagen könnte daher eine ganze Reihe neuer potenzieller Ziele für die Entwicklung von Wirkstoffen darstellen.
